# Horizontal Root Fracture Accompanied by Luxation of Coronal Fragment in a Maxillary Central Incisor: A Case Report

**DOI:** 10.5681/joddd.2013.039

**Published:** 2013-12-18

**Authors:** Maryam Gharechahi

**Affiliations:** Assistant Professor, Dental Research Center, Department of Endodontics, School of Dentistry, Mashhad University of Medical Sciences, Mashhad, Iran

**Keywords:** Central incisor, tooth dislocation, tooth fracture

## Abstract

Root fracture injuries affect up to 7% of permanent teeth. This type of injury is rarely seen in teeth with open apices and depending on the fracture site, the prognosis is good. This case report describes a horizontal intra-alveolar root fracture in the middle third of a maxillary central incisor associated with an extrusive luxation of the coronal segment and its treatment in a 6-year-old girl. The patient was observed under a regular follow-up regime. After 2 years, clinical examination showed normal tooth color and position, with a positive response to the pulp test.

## Introduction

Horizontal fractures of permanent teeth represent 0.5–7% of all dental injuries. Although this type of injury is rarely seen in teeth with immature root formation, the prognosis is generally good, depending on the fracture site.^1^ The most common type of root fracture is in the middle third of the root (57%), followed by the apical third (34%). Approximately 59% of untreated or splinted teeth maintain their vitality. Single fractures and those distant from the gingival level have better prognosis.^2^ The factors that determine the prognosis for the vitality of the pulp after tooth luxations also affect the prognosis for healing of a horizontal or transverse root fracture. Treatment is usually repositioning and stabilizing the coronal segment in its correct position and monitoring the tooth for an extended period for pulp vitality.^3^

## Case report

A 6-year-old girl was referred to the Endodontics Department of Mashhad Dental School. She had had a bicycle accident one hour previously.

Medical history showed no systemic disease or signs and symptoms of cerebral involvement. In extra-oral examination, no swelling, soft tissue injuries and asymmetry were found.

**Figure 1.**
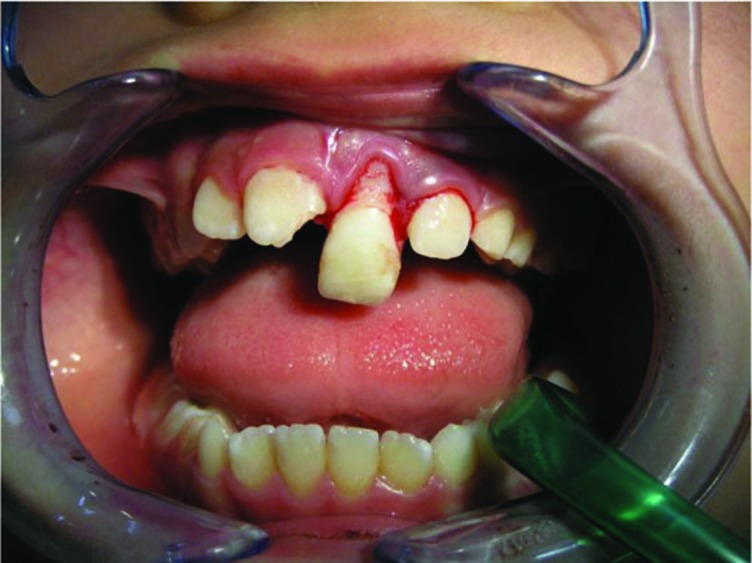
Clinical features of maxillary left central incisor.

**Figure 2.**
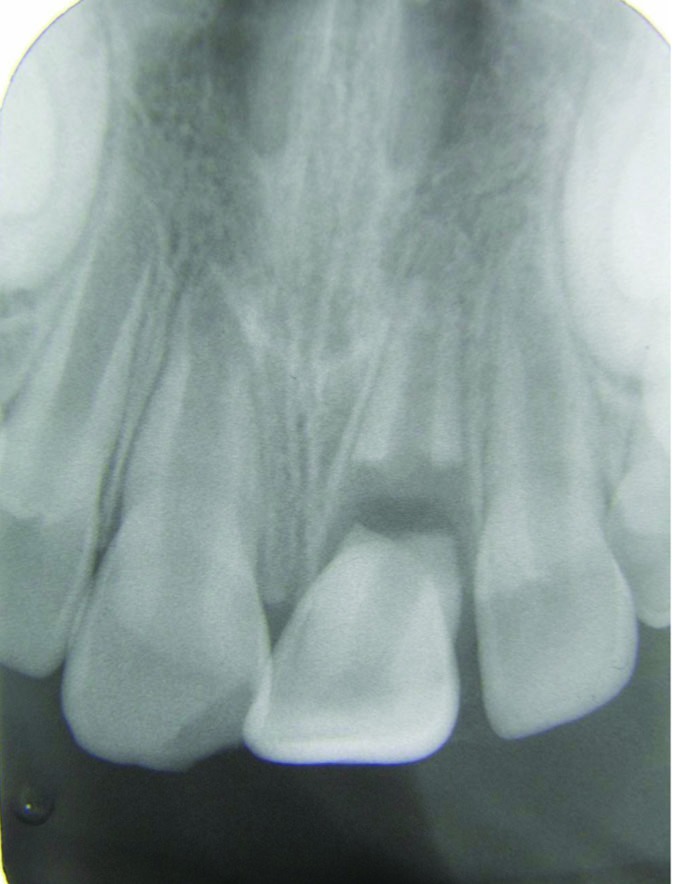
Radiographic image of the upper central incisors.

Dental clinical examination revealed significant mobility and extrusion of the maxillary left central incisor. The crown was extruded 4 mm and dislocated in a palatal direction (Figure 1). Fracture of the labial socket wall was not noticeable. The clinical picture of the case was consistent with a luxation injury; however, in radiographic examination, a horizontal fracture in the middle third of the root was seen. The upper right lateral incisor had an uncomplicated crown fracture (Figure 2).

**Figure 3.**
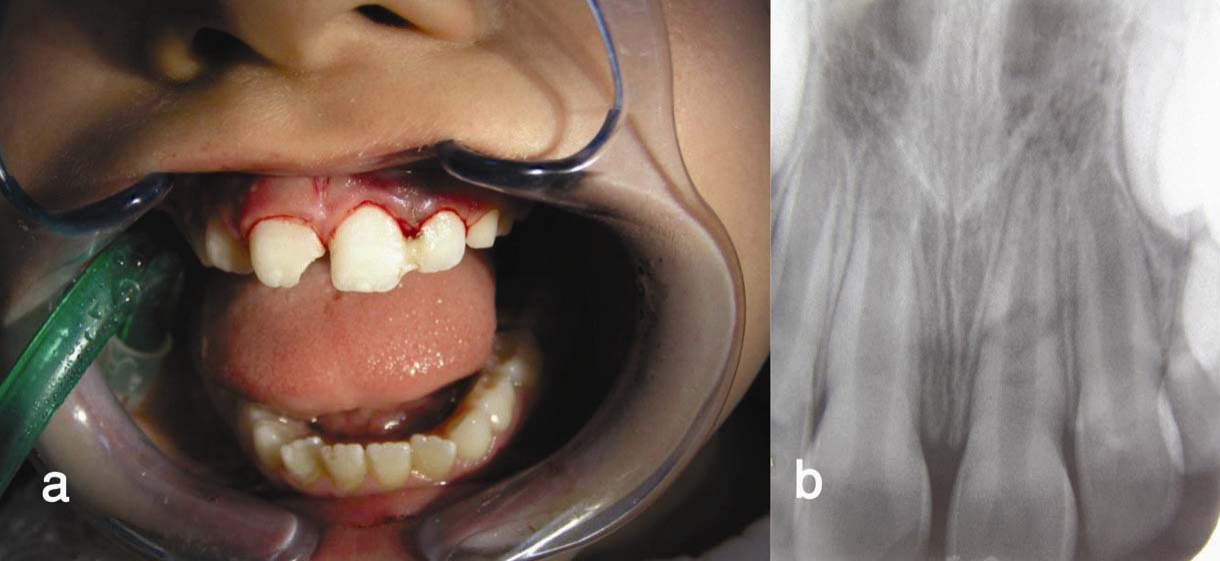
(a) The left central incisor was fixed to the left lateral incisor with composite resin; (b) the radiograph confirmed proper repositioning of the coronal fragment.

As the baseline value, all the mandibular and maxillary teeth underwent vitality tests. All the mandibular teeth responded to electric pulp tester (Vitality Scanner; Sybron Endo, Boston, MA) and cold test. The immediate treatment plan comprised reduction with gentle digital manipulation, repositioning and splinting of the coronal fragment under local anesthesia (2% lidocaine with 1:100000 epinephrine). Before splinting, for checking the optimal repositioning of the coronal fragment, the left central incisor was fixed to the left lateral incisor with composite resin (Figure 3) and a radiograph confirmed proper coronal fragment repositioning (Figure 3).

**Figure 4.**
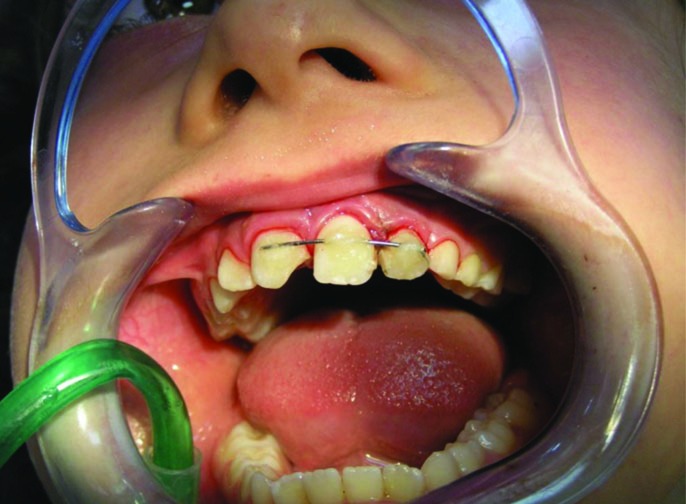
The splint was placed with stainless steel wire and fixed with light-cured composite resin.

After etching the labial surfaces of both maxillary central incisors and left lateral incisor, the bonding agent (Kuraray, Japan) was applied. Light-cured composite resin (Grandio, Voco, Germany) was applied to the etched enamel surfaces to support the stainless steel wire on the teeth (Figure 4). Antibiotics (amoxicillin), analgesics and chlorhexidine mouthrinse were also prescribed.

**Figure 5.**
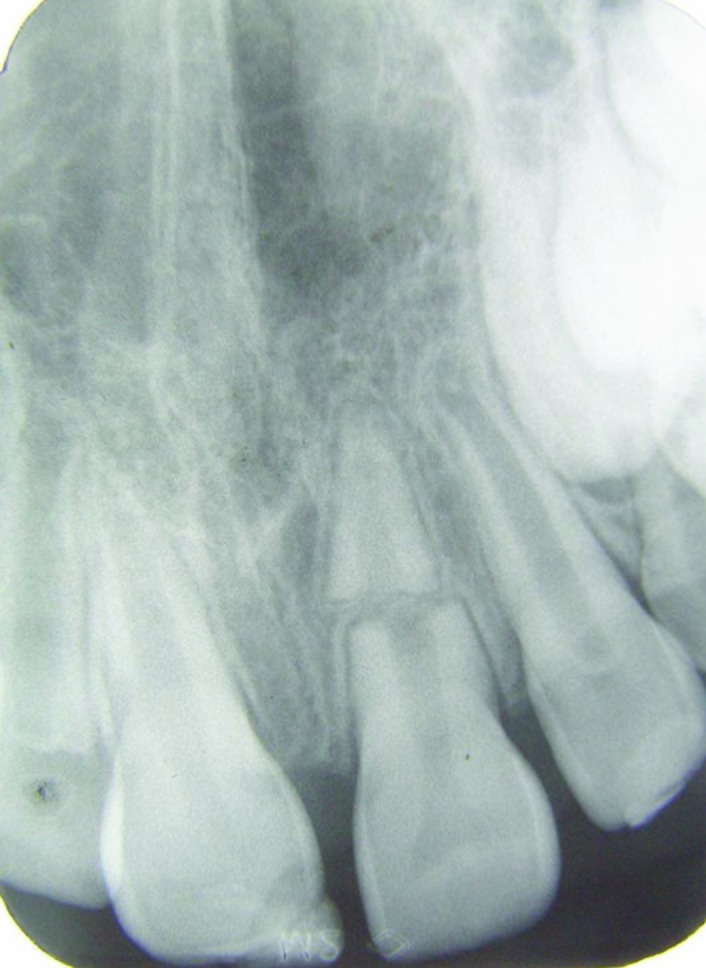
Radiographicimage of the upper central incisors after 3 months and removing the splint.

The splint was retained for 12 weeks. Radiographic and clinical examinations (sensitivity and percussion tests) were performed each month following repositioning. At the first two recall visits, the tooth did not respond to electrical and cold tests. However, the tooth was not tender to percussion and radiographic examination did not reveal any signs of developing pulp necrosis. Therefore endodontic intervention was not initiated. At the end of the third month the splint was removed (Figure 5) and the patient was scheduled for follow-up visits at 3-month intervals. It should be pointed out that during all the appointments, the vitality of other anterior maxillary and mandibular teeth was evaluated periodically.

**Figure 6.**
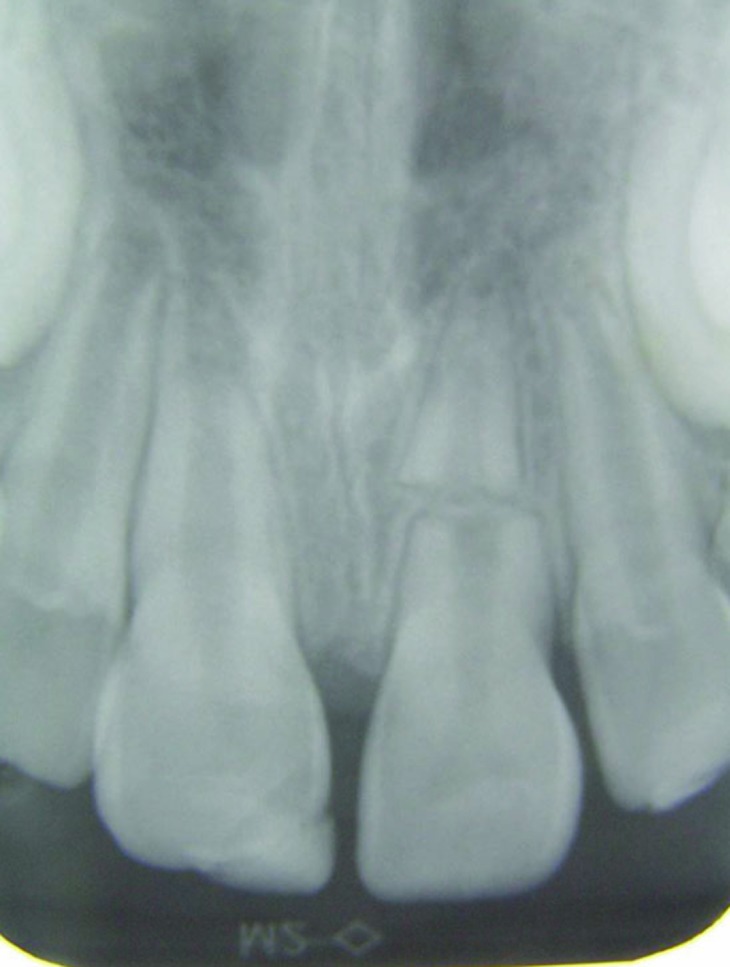
Radiographic image at 24-month follow-up, showing complete healing.

At the 24-month recall, the left central incisor had no mobility and pain in horizontal and vertical percussion tests. The crown was not discolored and the occlusion was normal. The tooth also responded normally to the electrical and thermal stimulation tests. In the radiographic examination, not only the thickening of dentinal walls of the root, but also the closure of apex, was noticeable. Healing of the fracture with calcified tissue was evident. External surface resorption (peripheral rounding), mesial and distal to the fracture site, which is a characteristic finding in horizontal root fracture, was detected radiographically (Figure 6).

## Discussion

Many factors may affect the type of healing which occurs in root fracture, including the time elapsed following trauma until presentation for treatment, the age of the patient, the stage of root development, the dislocation of the coronal fragment and any associated signs and symptoms, such as mobility and pain.^2^ In teeth with immature root formation healing with calciﬁed tissue is most often observed.^4^ Feely et al^2^ have affirmed a statistically signiﬁcant correlation between the stage of root development and the type of healing. They concluded that root-fractured teeth with immature roots had a better chance of healing than teeth with mature roots.

Pulp necrosis following root fractures occurs in 5–25% of the affected teeth.^5^ The risk of pulp necrosis is higher in mature teeth and those in which significant dislocation of the coronal fragment has occurred. A root-fractured tooth without displacement has a higher likelihood of maintaining its vitality than a displaced tooth.^6^ Andreasen et al^7^ showed that optimal repositioning of root fractures with dislocation of the coronal segment of up to 1 mm favored both healing with hard tissue and at the same time reduced the risk of pulp necrosis. In addition, it is generally accepted that roots with incomplete root formation have a greater potential for maintaining pulp vitality than those with closed apices.^5^

It has been reported that in up to 80% of cases healing of horizontal root fractures could take place with or without initial treatment.^8^ To treat root fractures, fixation for at least 2 to 3 months is recommended.^9^ The soft, round, 0.3-mm stainless steel wire offers stability, and its slight flexibility provides some physiologic mobility to the splinted teeth within the alveolar socket and developing occlusion.^10^

In this case, referral time was an important factor. The displacement of the coronal part was so severe that favorable healing would have been compromised if the tooth had not been repositioned at the initial examination. Along with appropriate management, careful follow-up of trauma cases is absolutely necessary. In this case, the incisor was immature with a wide root canal and an open apex that favored pulp survival. Potential regenerative properties of the pulp in young permanent teeth such as in this case are worth waiting to obtain better healing of the root fracture.

This case study demonstrated that dental traumas can be successfully resolved with minimal use of invasive techniques. The prognosis is generally good when treatment is performed as soon as possible following the injury. The present case illustrated favorable results by using suitable management technique resulting in hard tissue healing and maintenance of pulp vitality.

## Conclusion

Immediate treatment of the intra-alveolar root fracture with a severely displaced coronal fragment was important for good prognosis. Postoperative radiograph confirmed root fracture healing process after appropriate treatment by deposition of calciﬁed tissue between the fractured segments.
